# Anti-plasmodial and anti-inflammatory activities of cyclotide-rich extract and fraction of *Oldenlandia affinis* (R. & S.) D.C. (*Rubiaceae*)

**DOI:** 10.4314/ahs.v17i3.26

**Published:** 2017-09

**Authors:** Chukwuemeka Sylvester Nworu, Tochukwu Ifenyinwa Ejikeme, Adaobi Chioma Ezike, Okechukwu Ndu, Theophine Chinwuba Akunne, Collins Azubuike Onyeto, Paul Okpalanduka, Peter Achunike Akah

**Affiliations:** Department of Pharmacology & Toxicology, Faculty of Pharmaceutical Sciences, University of Nigeria, Nsukka, 410001, Enugu State, Nigeria

**Keywords:** *Oldenlandia affinis*, Reactive oxygen/nitrogen species, *Plasmodium berghei*, pro-inflammatory mediators, parasitemia

## Abstract

**Background:**

*Oldenlandia affinis*, commonly called ‘*kalata-kalata*’, a versatile plant used locally to treat malaria fever in some parts of sub-Saharan Africa was investigated for anti-plasmodial and anti-inflammatory activities.

**Objective:**

The study was designed to evaluate the antiplasmodial as well as anti-inflammatory activities of whole extract and cyclotide-rich fraction of *Oldenlandia affinis*

**Method:**

The dichloromethane-methanol extract (ODE) of the plant, *O. affinis* was investigated for suppressive and curative antiplasmodial activities against *Plasmodium berghei* in mice. ODE and the cyclotide-rich fraction (CRF) was investigated for chronic and acute anti-inflammatory activities in rat models of inflammation. Inhibition of pro-inflammatory mediators was studied in RAW264.7 macrophages.

**Results:**

ODE exhibited significant (p<0.05) reduction in mean parasitaemia in both the suppressive and curative models of *Plasmodium berghei* infection in mice.Administration of ODE(100, 200, or 400 mg/kg) and CRF (100, 200, or 400 mg/kg) produced significant inhibition of rodent models of acute and chronic inflammation . This observation is supported by the significant (P<0.05) inhibition of pro-inflammatory mediators, inducible nitric oxide (iNO) and tumour necrosis factor-alpha (TNF-α), and the reactive radical scavenging activities in RAW264.7 macrophages.

**Conclusion:**

These findings could explain, at least in part, the successes reported in the use of the herb, *Oldenlandia affinis* in the traditional treatment of malaria fever

## Introduction

Malaria is a global scourge and remains a leading cause of morbidity and mortality worldwide, especially in pregnant women and children, and particularly in tropical Africa. Sub-Saharan Africa carries a disproportionately high share of the global malaria burden. In 2015, the region was home to 88% of malaria cases and 90% of malaria deaths^1^. Despite the remarkable progress made in the fight against malaria, drug therapies are becoming less and less effective, due to drug resistance by *Plasmodium falciparum*. 2,3 Disappointed by apparent low effectiveness of orthodox anti-malarial treatments, and in search of relief, people living in malaria endemic countries have often resorted to the use traditional remedies either as a prophylactic measure to prevent frequent malaria episodes or to cure themselves^4^. This makes the investigation of these local remedies plausible and worth-while. It has been reported that more than 50% of new available drugs in the world today are still derived from natural sources.^5^ An important role in the challenge is to discover new anti-malarial drugs^6^.

*Oldenlandia affinis* (R. & S.) D.C. (*Rubiaceae*) is a scrambling perennial herb widespread in tropical Africa. During a Red Cross Relief Mission in Democratic Republic of Congo in the 1960s, Dr. Lorents Gran, observed that women in that region used ‘*kalata-kalata*’, a medicinal tea made from the leaves of the plant *Oldenlandia affinis* to induce labour and facilitate smooth and painless deliveries^7^. Later, in vivo studies in rats confirmed the uterotonic activity of *O. affinis* extract and the purified active ingredient, a peptide, named ‘*kalata B1*’, after the traditional name for the native medicine, *kalata-kalata*. Twenty years later, further research elucidated a cyclic cystine knot motif in its structure and identified the peptide as a cyclotide.^8^

In Benin Republic, *Oldenlandia affinis* is mixed with honey and used against learning impairment and the aqueous decoction used as anti-colic drink.^9^ Phytochemically, the plant has been shown to contain, apart from serotonin, several cyclotides with three cysteine-to-cystine bridges responsible for their special and compact structure, as well as their medicinal properties. Cyclotides, first isolated from *Oldenlandia affinis*, are small disulfide rich peptides isolated from plants.^10^ Typically containing 28–37 amino acids, they are characterized by their head-to-tail cyclised peptide backbone and the interlocking arrangement of their three disulfide bonds. These combined features have been termed the cyclic cystine knot (CCK) motif. To date, more than 100 cyclotides have been isolated and characterized; mostly from species of *Rubiaceae, Violaceae*, and *Cucurbitaceae* families.

Cyclotides are responsible for most of the therapeutic properties and potential of *O. affinis*. Cyclotides have been reported to possess a broad range of useful biological activities, including anti-HIV^11^, insecticidal^12^, anti-cancer^13^, anti-fouling^14^, anti-microbial^15^, anti-viral activity^16^, haemolytic, neurotensin antagonism^18^, trypsin inhibitory^19^, and uterotonic activities^11^,^14^,^20^.

In some rural communities in South-Eastern Nigeria, the decoction of *O. affinis* is used as anti-malarial and antifebrile remedy. This practice motivated this study in which we investigated the anti-inflammatory and anti-plasmodia activities of the whole plant extract and cyclotide-rich fraction of *O. affinis*. The whole aqueous methanol extract of *O. affinis* was investigated for preventive and curative anti-plamodial activities in mice infected with *Plasmodium berghei*. The effects of the extract against acute and chronic inflammatory responses were determined in rodent models and, in vitro, on the expression of inducible nitric oxide, TNF-α and the scavenging of reactive oxygen/nitrogen species (ROS/RNS) in cultures of monocytic cell line (RAW264.7).

## Materials and methods

### Collection of plant material and preparation of extract

The aerial parts of *Oldenlandia affinis* were freshly collected in the month of August, in Nsukka Local Government Area of Enugu State, Nigeria. The collected plant material was authenticated by a plant taxonomist, Mr. Alfred Ozioko of International Centre for Ethnomedicine & Drug Development (InterCEDD) Nsukka, Enugu Statewhere a voucher specimen (InterCEDD/994) has been deposited. The plant material was washed in stream of clean water and contaminating debris removed, thereafter air-dried at room temperature, and then pulverised. A portion of the powdered plant (700 g) was macerated in 6 L of a 1:1 water and methanol for 48 h with intermittent agitations; the resultant mixture was filtered through Whatman (No. 1) filter paper and the filtrate concentrated in vacuo at 40°C using rotary evaporator. This whole aqueous-methanol extract of *O. affinis* aerial parts (ODE) was as stored at −20 °C for future use.

The cyclotide-rich fraction was prepared using a previously reported protocol^21^. Briefly, the whole aerial part extract (ODE) was enriched for cyclotide by partitioning repeatedly (3x) between an aqueous phase and equal volume of dichloromethane in a separating funnel. The organic soluble fraction was discarded, and the aqueous layer, which contains the cyclotides as reported earlier^22^, was concentrated using a rotary evaporator. Freeze-drying of the concentrated fraction yielded a dark-brown extract, a cyclotide-rich fraction (CRF), which was stored in aliquots at −20°C and used subsequently for the experiments.

Preliminary phytochemical tests were carried out on both the aqueous-methanol extract and on the cyclotide-rich fraction using the procedures outlined by Harborne^23^.

### Animals

Adult Swiss albino mice (18–25 g, 7–8 weeks old) of both sexes were procured and housed in the Laboratory Animal Facility of the Department of Pharmacology and Toxicology, University of Nigeria (Nsukka). The animals were housed under standard conditions 25 ± 2°C, relative humidity of 50 ± 5%, and a 12-h light/dark cycle,) and had access to standard rodent chow pellets (Livestock Feed PLC, Lagos, Nigeria) and unrestricted access to drinking water. All mice were allowed at least 5 days to acclimatize prior to use in the studies. The use and care of the animals were in accordance with ethical guidelines as contained in the European Union Directives for the Protection of Animals used for Experimental and other Scientific Purposes (EU Directive: 2010/63/EU) of 2010 which conforms with local institutional ethical guideline.

### Median Lethal Dose (LD_50_) on ODE and CRF

The Median Lethal Dose (LD_50_) of the ODE and CRF (of ODE extract) was estimated in mice by the oral route using the method of Lorke^24^. The tests involved two phases: the first involved determination of the toxic range. The mice were placed in groups (n = 3) and ODE or CRF (10, 100, or 1000 mg/kg) was administered intraperitoneally (i.p). The treated mice were then monitored for 24 h for mortality. In the second phase, four different doses of ODE or CRF were administered orally based on the earlier outcomes. The mice were observed for lethality and signs of acute intoxication for 24 h. The LD_50_ was then calculated as the geometric mean of the highest non-lethal dose and the least toxic dose^24^.

### Studies on anti-plasmodial effects of ODE

#### Maintenance of *plasmodium* parasites and parasite inoculation

Sample of red blood cells parasitized by *Plasmodium berghei* was obtained from a donor-infected mouse maintained at Animal Facility Centre, Faculty of Veterinary Medicine, University of Nigeria Nsukka. Parasite was maintained by continuous re-infestation in mice. Parasitized mouse blood was diluted in sterile saline to obtain a 5x10^7^ parasitized erythrocytes/ml. Mice used in the study were inoculated intra-peritoneally with 0.2 ml of infected blood suspension containing 10^7^ *P. berghei* parasitized red blood cells using a 25G needle. Prior to inoculation of recipient mouse, parasitemia in the donor mouse was ascertained by microscopic examination of Giemsa-stained thin blood smear.

#### Evaluation of Suppressive Anti-plasmodial activity of ODE in infected Mice

Suppressive/prophylactic assay was used to evaluate the in vivo anti-malarial activity of *O. affinis* whole aerial part extract (ODE) against *P. berghei* infection using the method described by Knight and Peters^25^. Twenty five Swiss albino mice of either sex (18–25 kg) were infected by intraperitoneal inoculation of 10^7^ *P. berghei*- parasitized red blood cells in 0.2 ml volumes. The mice were randomly assigned to five groups (n=5) and pre-treated(intraperitoneally, i.p.) with either normal saline (10 ml/kg/day) to serve as the negative control group, a standard anti-malarial-artemether (100 mg/kg/day), or ODE (100, 200, or 400 mg/kg/day). ODE and the controls were administered from day zero through day 4. On day 5, thin blood smears were prepared from tail blood of each mouse, fixed in absolute ethanol and stained with 10% Giemsa stain. The stained smears were examined microscopically (×100 magnification) and the number of parasitized RBC counted in 10 different fields determined. Then the degree of parasitaemia (%) was calculated by:

$$Parasitaemia\;(\%)=\frac{Number\;of\;parasitised\;RBCs\;in\;fields}{Total\;Number\;of\;RBCs\;in\;fields}*100$$

The percentage inhibition of parasitaemia was also calculated according to the formula:

$$Inhibition\;of\;parasitaemia\;(\%)=100-\left(\frac{\;\%\;parasitaemia\;of\;treated\;group}{Mean\;\%\;parasitaemia\;of\;control\;group}\right)*100$$

Levels of parasitaemia in the treated groups were then statistically compared to the negative control.

#### Evaluation of curative anti-plasmodial activity of ODE on established *plasmodium berghei* infection in mice

To evaluate the curative anti-malarial properties of ODE on established *Plasmodium berghei* infection, twenty five Swiss albino mice were each inoculated intraperitoneally with 1 × 10^7^ *P. berghei*-parasitised RBCs in 0.2 ml volumes on day zero^26^. Seventy-two hours later, the mice were assigned to five groups (n=5) and were treated once daily (i.p) (intra-peritoneally) with either 10 ml/kg normal saline (negative control), artemether (100 mg/kg; standard drug) and with three doses of ODE 100, 200, and 400 mg/kg) for 5 days. To determine the daily parasitaemia level, about three drops of blood were collected from the tail of each mouse and smeared onto a microscope slide to make a thin film. The smears were fixed with absolute ethanol and stained with 10% Giemsa stain, and examined microscopically (×100 magnification). The stained smears were examined microscopically (×100 magnification) and the number of parasitized RBC counted in 10 different fields determined. Then the degree of parasitaemia (%) was calculated by:

$$Parasitaemia\;(\%)=\frac{Number\;of\;parasitised\;RBCs\;in\;fields}{Total\;Number\;of\;RBCs\;in\;fields}*100$$

#### Effect of ODE and CRF on carrageenan-induced acute paw inflammation in rats

The method described by Winter et al.^27^ was adopted. Twenty-five Swiss albino rats were randomly divided into 5 groups (n=5). Each of the animals in Group I received 0.2 ml of normal saline (intra-peritoneally) and served as the negative control group while animals in groups II were administered 50 mg/kg of ODE; Group III received 100 mg/kg of ODE and Group IV received 200 mg/kg of ODE. Animals in Group V received piroxicam (50 mg/kg; i.p) and served as the reference standard group. The groups are similar inseparate studies, but Group II-IV were administered CRF (50, 100 or 200 mg/kg; intraperitoneally) respectively. One hour after administration of drugs and extract, 0.1 ml of sterile saline solution of 1% carrageenan was injected into the sub-plantar surface of the left hind paw. Paw size was studied by measuring the volume of water displaced by the inflamed paw at time 0 h (before carrageenan administration) and at time 0.5, 1, 2, 4, 6 and 24 h after the carrageenan administration. The percentage inhibition of paw volume in treated groups was compared with the negative control group.

The ability of test agents to suppress paw inflammation was expressed as a percentage of inhibition of paw oedema and calculated according to the following equation. 28

Inhibition (%) = (C-T) / C x 100

Where C = Mean increase in paw thickness of control group and T = Mean increase in thickness of treatment group.

#### Effect of ODE and CRF on formaldehyde-induced_model of chronic inflammation in rats

rat-model of chronic inflammation was assessed utilizing formaldehyde-induced paw oedema^29^. Adult Swiss albino rats of both sexes were grouped into five groups (n=5). Group I animals each received 0.2 ml of normal saline (intraperitoneally) and served as the negative control group. Animals in groups II to IV received ODE (100, 200 & 400 mg/kg; i.p) or CRF (100, 200 & 400 mg/kg; i.p) respectively. Animals in Group V received dexamethasone (1 mg/kg; intraperitoneally) and served as the reference standard group. One hour later, inflammation was induced by sub-plantar injection of 0.1 ml of 2% formaldehyde solution and repeated on day 3, while drug administration was continued from day 1 to day 10. Daily changes in oedema were evaluated by measuring the volume of water displaced by the inflamed paw once daily for the 10 days.

The percentage inhibition of paw volume in treated groups was compared to the negative control group. The ability of test agents to suppress paw inflammation was expressed as a percentage of inhibition of paw oedema and calculated according to the following equation 28.

#### Inhibition (%) = (C-T) / C x 100

Where C = Mean increase in paw thickness of control group and T = Mean increase in thickness of treatment group.

### In vitro experiments

#### Cell lines and culture conditions

RAW264.7 monocytic cell line was cultured in R-10 (RPMI 1640 medium; Mediatech Inc., Manassas, VA) supplemented with 10% heat-inactivated Foetal bovine saline (FBS), 50 µM 2-mercaptoethanol, 100 IU penicillin/ml, and 100 µg streptomycin/ml (Gibco, Grand Island, NY).

### Effects of CRF on cell viability

Effects of CRF on RAW264.7 cell viability were evaluated using a modification of the original MTT (3-(4,5-dimethylthiazol-2-yl)-2,5-diphenyltetrazolium bromide) viability assay of Mosmann 30. RAW 264.7 cells were cultured for 24 h in 96-well flat-bottom plates at 5 × 10^4^ cells/well at 37°C 5% CO_2_ chamber. Cells were then treated with graded concentrations of CRF (5, 25, 50, 100, 250, or 1000 µg/ml) or 1 µg lipopolysaccharide/ml (LPS; serotype 0128:B12, L 4255; Sigma) as a standard mitogen. The culture plate was then incubated for 48 h at 37°C. At the end of this period, 20 µl MTT (5 mg/ml in phosphate buffered saline; PBS) was added to each well and the plates incubated for 4 h at 37°C. The plates were then centrifuged and culture medium gently discarded. Formazan crystals formed in viable cells were dissolved by addition of 150 µl DMSO/well and the optical density (OD) in each well was then read at 570 nm in a Bio-Kinetic Reader-E312e® plate reader (Bio-Tech Instruments, Winooski, VT). The proliferation index (PI), reflecting cell viability, was calculated as Mean ODtest/Mean ODcontrol.

### Effect of CRF on LPS-evoked iNO production by RAW264.7

Nitric oxide production and release by RAW264.7 cells was measured indirectly by determining nitrite accumulation in culture supernatant using the Griess reaction. RAW264.7 cells (1x10^5^ cells/well) were seeded in 96-well flat bottom for 24 h. The cells were pre-treated for 2 h with CRF (0, 5, 25, 50, or 100 µg/ml) before LPS (1 mg/ml) (serotype 0128: B 12, L 4255; Sigma, St Louis, MO) was added to the wells. After incubation, cell-free supernatant was harvested and analysed. When analysis was not possible on the day of collection, the supernatant was stored at −20 °C and analysed subsequently after thawing at room temperature. In the assay for nitrite levels, 100 ml of cell-free culture supernatants were mixed with an equal volume of freshly prepared Griess reagent in 96-well plate and incubated at room temperature for 10 min. The absorbance was measured on a microplate reader at 570nm (Bio-Kinetic Reader-E312e®; Bio-Tech Instruments, Winooski, VT). Nitrite concentrations were extrapolated from a standard NaNO_2_ curve included within each assay plate.

### Effect of CRF on LPS-evoked tumor necrosis factor (TNF)-α production/release by RAW264.7

The effect of CRF on LPS-induced release of TNF-α, a pro-inflammatory cytokine by macrophages was determined. RAW264.7 cells 1x10^5^ cells/well) were seeded in 96-well flat bottom plate for 24 h. The cells were pre-treated for 2 h with CRF (0, 5, 25, 50, or 100 µg/ml) before LPS (1 mg/ml) was added to the wells. After incubation, cell-free supernatant was harvested and analysed. When analysis was not possible on the day of collection, the supernatant was stored at −20 °C and analysed subsequently after thawing at room temperature. The level of TNF-α in each supernatant was measured using a commercial ELISA kit (Mouse TNF-α DuoSet®, R&D Systems, Minneapolis, MN) following manufacturer instructions. The concentration of TNF-α was calculated from a standard curve of the standard mouse TNF-α included ELISA assay. The level of sensitivity of the kit was 31.2 pg TNF-α /ml.

### Effect of CRF on ROS and RNS by enhanced-luminol chemiluminescence

Chemiluminescence of RAW264.7 macrophages was evaluated in Hank's balanced salt solution (HBSS; pH 7.4) by a modification of the enhanced-luminol microplate assay^31^,^32^. The principle of the method is based on a luminol interaction with the phagocyte-derived ROS and RNS, which results in large measurable amounts of light.^30^ RAW264.7 cells were seeded 2x10^5^ cells/well into a 96-well plate (white opaque- walled). The plate with treated with CRF (0, 5, 25, or 50 µg/ml) and incubated for 30 min. Enhanced luminol solution (Pierce Biotechnology Inc., Rockford, IL) was added into the well and placed in a microplate luminometer (Synergy H1 Hybrid Multi-Mode, BioTek Winooski, VT) and luminescence read for 5 min. Thereafter, a solution of phorbol 12-myristate 13-acetate (PMA) was delivered automatically into each well to achieve a final concentration of 1 mM. Light emission expressed as relative light units (RLU) was recorded continuously at 37 °C for 60 min following PMA stimulation. Intensity of the chemiluminescence reaction was expressed as the integral of the obtained kinetic curves which corresponded to the total amount of light produced during the time of measurements. Luminescence data was analysed and integrated using Gen 5 version 2.0 (BioTek, Winooski, VT) and expressed as RLU.

### Statistical analysis

All results were expressed as mean [± SEM]. Data were analyzed with Graphpad Prism™ (version 5.0) by a oneway analysis of variance (ANOVA) followed by a Bonferroni's post-hoc test. Differences between mean observations were considered significant at p-values ≤ 0.05.

## Results

### Phytochemical analysis and extractive yield

The yield of the crude Oldenlandia affinis extract (ODE) was determined to be 204.7 g (5.12%, w/w), while that of the cyclotide-rich fraction (CRF) was 41.5 g (5.93%, w/w). Preliminary phytochemical studies on ODE and CRF showed the presence of a variety of bioactive phytoconstituents in different amounts. Saponin, tannins, reducing sugar, glycosides, proteins, acidic compounds, alkaloids and flavonoid were found to be present in ODE while flavonoids, proteins and alkaloids were relatively more abundant in CRF; steroids and terpenoids were also present in CRF (Table 1).

**Table 1:** Phytochemical analysis of extract and fraction

Constituents	Crude extract (ODE)	Cyclotide-rich fraction (CRF)
**Flavonoids**	+++	+++
**Glycosides**	−	−
**Alkaloids**	+++	++
**Saponins**	−	++
**Tannins**	+	−
**Proteins**	+++	+++
**Reducing sugars**	+	++
**Steroids**	++	+
**Terpenoids**	++	−
**Fats & oils**	−	−

(Where − = absent; + = present in low amounts; ++ = moderately present and +++ = abundantly present).

### Acute toxicity

Oral administration of up to 5000 mg/kg of both the extract ODE and its cyclotide rich fraction (CRF) to mice did not cause lethality in the two stages of this test. The LD_50_ of both extract and fraction was therefore estimated to be greater than 5000 mg/kg^24^. Similarly, the animals did not exhibit signs of acute intoxication during the period of observation.

### Prophylactic Activities of ODE on *P. berghei* Infection in Mice.

**Figure 1:**
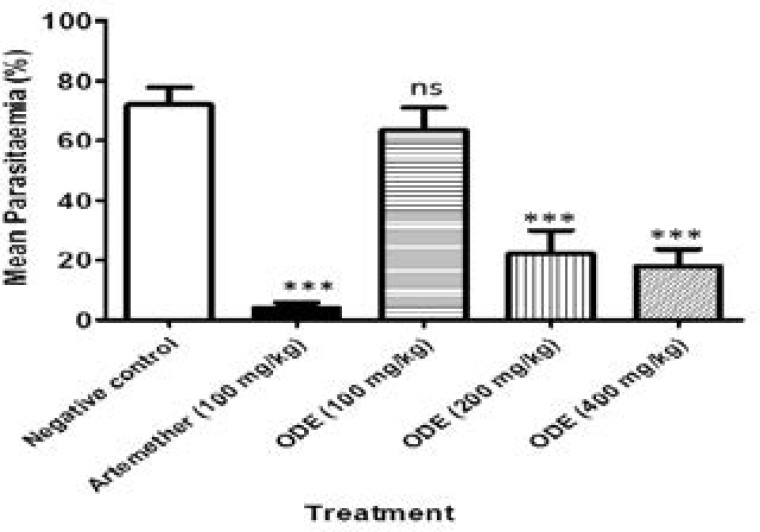
Prophylactic effects ODE on *Plasmodium berghei* infection in mice. ***P<0.001, ns = not significant, compared to vehicletreated group (One-way ANOVA followed by Bonferroni's post hoc test).

ODE exhibited significant (p<0.05) suppressive activity against *Plasmodium berghei* in vivo at all of the three doses tested (Figure 1), seen as reduction in mean percentage parasitaemia on the 5th day post infection, compared to the untreated control group. Administration of ODE (100, 200, and 400 mg/kg) reduced percentage parasitaemia from 72.0±5.7% to 63.5±7.5%, 22.2±7.7, and 18.0±5.7 representing 11.8%, 69.1, and 75% reductions in parasitaemia, respectively, when compared to the untreated control group. Similarly, artemether (100 mg/kg) reduced parasitaemia to 4.2±1.7% (a 94.1% reduction versus control).

### Curative activities of ODE on *P. berghei* Infection in Mice.

**Figure 2:**
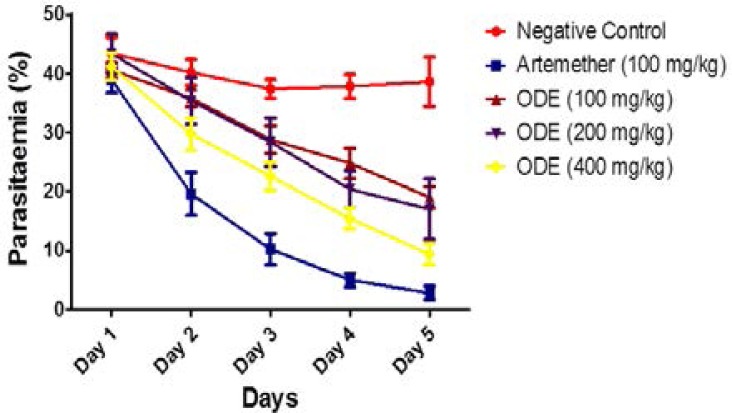
Time-course effect of ODE on percentage parasitaeia of established *Plasmodium berghei* infection in mice. Data is presented as mean ± SEM. ^a^P<0.001, ^b^P<0.01 ; ^c^P<0.05 compared to vehicle-treated group (two-way ANOVA followed by Bonferroni's post hoc test).

ODE reduced the parasitaemia significantly (P<0.05) from the first day of treatment and achieved the highest effect on the 5^th^ day (Figure 2). At the dose of 100 mg/kg, administration of ODE produced 6.5%, 17.5%, 33.6%, 42.9%, and 56.2% reductions in parasitaemia from day 1 to day 5 after treatment, respectively. For the group of mice that received 200 mg ODE/kg, the mean reductions in parasitaemia were in order of 0.0%, 18.43%, 34.56%, 53%, and 60.8% from day 1 to day 5 after treatment, respectively. Similarly, the mean percentage parasitaemia decreased in the groups that received 400 mg ODE/kg in the order 5.1%, 31.3%, 47.9%, 64.5%, and 78.3% from day 1 to day 5 after treatment, respectively (Figure 2). The group that was treated with the standard antimalarial drug, artemether (100 mg/kg) produced mean percentage parasitaemia reductions of 10.1%, 54.84%, 76.5%, 88.5%, and 93.6% from day 1 to day 5 post treatment, respectively, (Figure 2). When compared, the mean percentage parasitaemia suppression produced in the group of infected mice that received the standard drug, artemether, is not statistically different (P>0.05) from mean percentage suppression in the ODE (400 mg/kg) group. The highest dose of ODE (400 mg/kg) produced a maximum 78.3% parasitaemia suppression on the 5^th^ day while artemether produced a parasitaemia suppression of 93.6% on the 5^th^ day.

### The effect of the crude extract (ODE) on carrageenan-induced paw oedema in rats

On assessing the acute anti-inflammatory activity with this model, treatment with both the crude extract (ODE) and the standard agent, piroxicam, significantly reduced the paw oedema induced by carrageenan. In the negative control group, carrageenan produced a local paw oedema, reaching its maximum at 6 h (Table 2). Administration of the crude extract (ODE) at a dose of 100 mg/kg, produced the greatest significant inhibition of 78.26% at 6 h (P<0.001) compared to negative control. The standard group (piroxicam 50 mg/kg) also produced significant reduction in paw oedema; exhibiting 100% inhibition at 2 h compared to the control (P<0.001).

**Table 2:** The effect of ODE on carrageenan-induced paw oedema in rats

Treatment	Paw volume (ml)					
	0 h	0.5 h	1 h	2 h	4 h	6 h
**Negative** **control (normal saline)**	0.36±0.02	0.46±0.02	0.58±0.06	0.78±0.06	0.78±0.06	0.82±0.06
**ODE 50 mg/kg**	0.36±0.02	0.44±0.04 (20%)	0.44±0.03* (63.64%)	0.50±0.03*** (66.67%)	0.54±0.04*** (57.14%)	0.50±0.00*** (69.57%)
**ODE 100 mg/kg**	0.34±0.02	0.44±0.02 (0%)	0.46±0.02* (45.45%)	0.46±0.02*** (71.43%)	0.46±0.02*** (71.43%)	0.44±0.02*** (78.26%)
**ODE 200 mg/kg**	0.36±0.02	0.48±0.02 (−20%)	0.52±0.02 (27.27%)	0.6±0.05 (42.86%)	0.54±0.02*** (57.14%)	0.52±0.02*** (65.22%)
**PIROXICAM50mg/kg**	0.36±0.02	0.48±0.04 (−20%)	0.40±0.03** (81.82%)	0.36±0.02*** (100%)	0.40±0.00*** (90%)	0.38±0.02*** (95.65%)

Values are presented as mean ± SEM. Percentage inhibition given in parenthesis.

* P<0.05;

** P<0.01;

*** P<0.001 compared to negative control.

### The effect of extract (ODE) on formaldehyde-induced inflammation in rats

The anti-inflammatory effect of the crude extract (ODE) in this formaldehyde-induced chronic arthritis model in rats is shown in the Table 3 below. Both crude extract and standard drug (Dexamethasone) significantly attenuated progression of chronic inflammation induced by formaldehyde (P<0.05) compared to negative control. The greatest level of inhibition was exhibited by ODE at 400 mg/kg, producing a significant inhibition of 94.4% on Day 9 in this model. The standard group (Dexamethasone 1 mg/kg) also showed significant inhibition in oedema exhibiting 77.6% inhibition (P<0.01) on Day 10.

**Table 3:** The effect of ODE and CRF on formaldehyde-induced chronic arthritis in rats

Treatment	Dose (mg/kg)	Chronic inflammation index (measured as AUC of global oedema in cm^−3^. hr)	Inhibition of chronic inflammation (%)
**Negative control** **(normal saline)**		391.92±22.32	____
**Dexamethasone**	1	269.28±18.48	31.29±4.72
**ODE**	100	357.12±28.56	8.88±7.29
	200	288.96±22.56	26.27±5.76
	400	280.32±10.8	28.48±2.76
**CRF**	100	279.12±26.88	28.78±6.86
	200	246.72±12.28	37.05±3.12
	400	279.84±16.80	28.60±4.29

Values are presented as mean ± SEM. Percentage inhibition given in parenthesis.

*P<0.05; **P<0.01; ***P<0.001 compared to negative control

### Effect of crf on viability of raw264.7 cells

**Figure 3:**
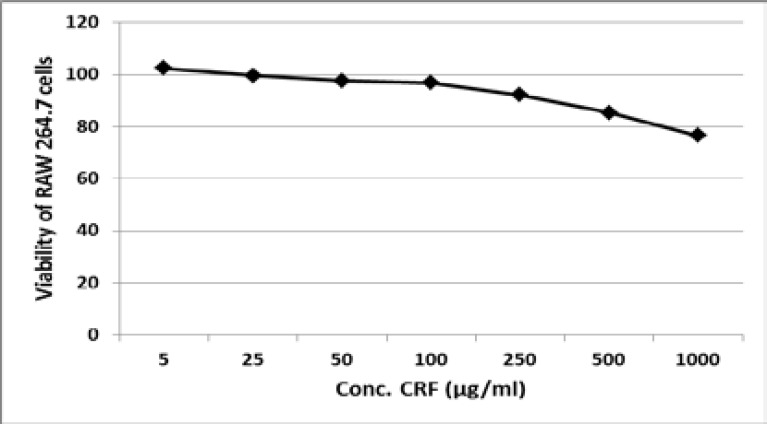
Effect of CRF on viability of RAW264.7 cells

Treatment of RAW264.7 with 5–1000 µg CRF/ml for 48 h showed that the cells remain largely viable at concentration less equal to or 250 µg/ml (Figure 3). The viability of the RAW264.7 cells was as high as 96.87% at 100 µg CRF/ml. However, cell viability was reduced to 85.43% and 76.54% following exposure to 500 and 1000 µg CRF/ml, respectively.

### Effect of CRF on LPS-induced nitric oxide production in RAW264.7 culture

**Figure 4:**
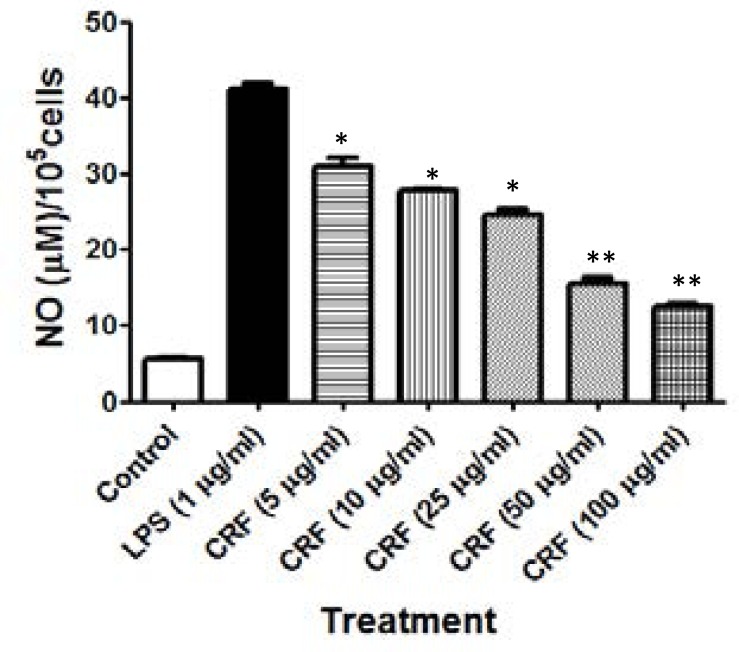
Effect of CRF on LPS-induced nitric oxide production in RAW264.7 culture. **P<0.01 ; *P<0.05 versus LPS-alone treated cells

The effect of the CRF on the release of nitrite from LPS-induced RAW264.7 cells is shown in Figure 4.

LPS-stimulated iNO expression was inhibited in a concentration-related manner when the RAW264.7 cells were treated with CRF (5–100 µg/ml). Pre-treatment of RAW264.7 cells with graded doses of CRF significantly (P<0.05) inhibited LPS-induced iNO levels in the culture supernatant in a concentration-related manner. Stimulation of RAW264.7 cells with LPS (1 µg/ml) evoked a level of iNO up to 41.12[±0.82] µM compared to the iNO level of 5.64[±0.19] µM measured in the supernatant of unstimulated culture. However, pretreatment with CRF (5, 10, 25, 50 and100 µg/ml) significantly (P<0.05) inhibited the production of inducible nitric oxide from mean level of 41.12[±0.82] µM in culture wells treated with LPS alone to 30.9[±1.10], 27.76[±0.23], 24.54[±0.83], 15.49[±0.75] and 12.47[±0.43] µM; representing an inhibition of 28.81 [±3.11], 37.66[±0.65], 46.73[±2.33], 72.24[±2.12] and 80.75[±1.21] %, respectively. The iNO median inhibitory concentration (IC_50_) of CRF in RAW264.7 is estimated as 49.53 µg/ml.

### Effect of CRF on LPS-evoked TNF-α production in RAW264.7 culture

**Figure 5:**
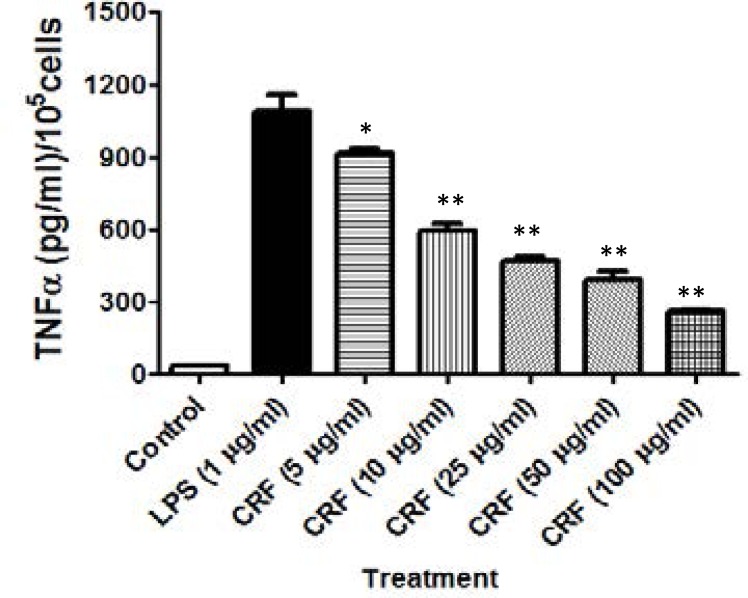
Effect of CRF on LPS-evoked TNF-α production in RAW264.7 culture. **P<0.01 ; *P<0.05 versus LPS-alone treated cells

The effect of the CRF on the production of a proinflammatory cytokine, TNF-α from LPS-induced RAW264.7 cells is shown in Figure 5.

LPS-evoked release of TNF-α was inhibited in a concentration-dependent manner when the RAW264.7 cells were treated with CRF (5–100 µg/ml). Pre-treatment of RAW264.7 cells with graded doses of CRF significantly (P<0.05) inhibited LPS-induced iNO levels in the culture supernatant in a concentration-related manner. Stimulation of RAW264.7 cells with LPS (1 µg/ml) induced a high level of TNF-α of 1093.00[±63.6] pg/ml compared to the TNF-α level of 34.75[±3.20] pg/ml measured in the supernatant of unstimulated culture. However, pretreatment with CRF (5, 10, 25, 50 and100 µg/ml) significantly (P<0.05) diminished the TNF-α production from the mean value of 1093.00[±63.6] pg/ml in LPS treated wells to 916.70[±17.64], 593.30[±29.63], 470.00[±15.28], 393.30[±29.63], and 260.00[±8.66] pg/ml; representing an inhibition of 16.66[±1.67], 47.22[±2.80], 58.87[±1.44], 66.12[±2.80] and 78.71[±0.82] %, respectively. The TNF-α median inhibitory concentration (IC_50_) of CRF in RAW264.7 is estimated as 50.17 µg/ml.

### Inhibition of ROS and RNS induced in PMA-stimulated RAW264.7 cells by CRF

**Figure 6:**
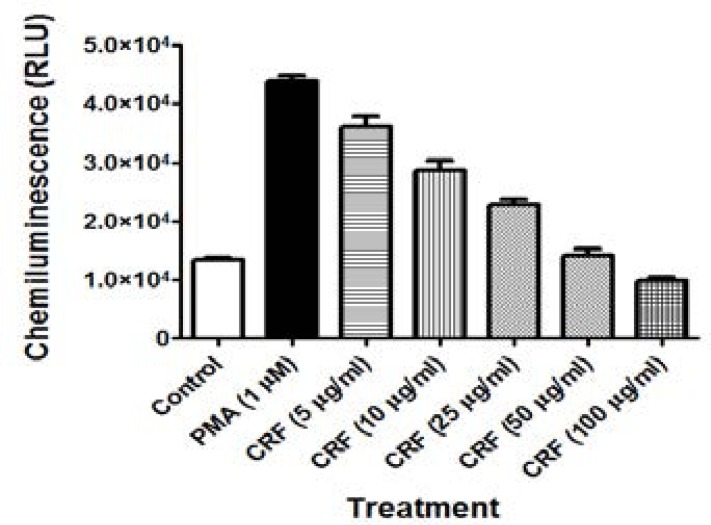
Inhibition of reactive oxygen species (ROS) and reactive nitrogen species (RNS) induced in PMA-stimulated RAW264.7 cells by CRF

Changes in production of ROS/RNS were determined by enhanced-luminol chemiluminescence assay. Pre-treatment of RAW264.7 macrophages with CRF (5, 10, 25, 50 and100 µg/ml) followed by stimulation with PMA (1 mM) caused significant (P<0.05) and concentration-dependent suppression of chemiluminescence detected and recorded over a 60 min period by a microplate luminometer (Figure 6). At 50 and 100 µg/ml, the chemiluminescence of luminol evoked by ROS/RNS was inhibited by as much as 72.24 [±2.12] and 80.75 [±1.21] %, respectively by CRF.

## Discussion

The centrepiece in the global treatment and control of malaria for over a decade has been the Chinese herb, Artemisia annua-derived artemisinin and the artemisinin-based combination therapy (ACT). The ACT is recommended by the World Health Organization as firstline treatment of uncomplicated *Plasmodium falciparum* malaria in sub-Saharan Africa.^33^ The combination comprises a fast-acting artemisinin derivative with rapid effect on parasite clearance, and a long-acting drug to prevent recrudescence and development of resistance. Despite huge success recorded with the use of ACT, there are increasing case reports of failure and resistance.^34^–^38^ The increasing cases of failures and resistance to ACT is a major motivation and incentive for increased research efforts aimed at getting a potent and cost-effective alternative treatment against the menace of *Plasmodium falciparum*.

Historically, many successful anti-malarial treatments such as quinine and artemisinin have been derived from medicinal plants. This research is partly motivated by the belief that it is still possible to obtain successful antimalarial molecules or lead molecules from medicinal plants, especially from herbs used by locals in the treatment of malaria and malaria fever. This present research was designed to evaluate cyclotide-rich extract and fraction from *Oldenlandia affinis*, a herb with versatile application in traditional folklore medicine. The local use of herbal teas and decoctions of *O. affinis* for the treatment of malaria, malaria fever and other diseases which are associated with inflammation inspired this study in which the prophylactic and curative anti-plasmodial activities, as well as anti-inflammatory and reactive radical scavenging properties of cyclotide-rich extract and fraction of *Oldenlandia affinis* were studied.

*Oldenlandia affinis* belongs to the *Rubiaceae* family of medicinal plants. Previous studies have shown that *O. affinis* is rich in cyclotides. Cyclotides belong to a family of special proteins characterized by their head-to-tail cyclic backbone and a cystine knot arrangement of three conserved disulfide bonds.^39^ They are remarkably stable and are characterized by a variety of bioactivities. It is believed that their natural presence is to serve as a defensive mechanism, protecting the plants in which they are found from pest or pathogens.^10^ The circular protein backbone together with the knotted arrangement of disulfide bonds is unique structural motif and confers cyclotides exceptionally stability to heat and resistance to proteolysis.^40^ The stability of cyclotides make them very attractive scaffold for drug development and other pharmaceutical applications.

In the prophylactic anti-plasmodial assay, prior treatment with the extract of *O. affinis*, ODE significantly reduced parasitaemia following *Plasmodium berghei* infection. The 4 day suppressive test is a standard test commonly used for anti-malarial screening.^41^ The extract produced significant dose related parasitaemia suppression in all the treated groups with parasitaemia suppression as high as 69.1% and 75% observed in the groups treated with 200 mg and 400 mg ODE extract/kg. The extract also showed significant dose related reduction in parasitaemia in established (curative) *Plasmodium berghei* infection, slightly lower than the effect of artemether, which in this study was used as standard control drug.

The results of the anti-plasmodial tests showed that the extract is active against the *P. berghei* malaria parasite used in this study and is consistent with the ethnomedicinal use of *Oldenlandia affinis* herbal decoctions and tea in the traditional treatment of malaria and malaria-related fever in south eastern Nigeria. *Plasmodium berghei* rodent model of plasmodial infection has been shown to produce disease features that are similar to those of human malaria infection.^42^–^44^ In this model, substances that reduce parasite multiplication (anti-plasmodial effect) in the host were considered to possess anti-malarial activity.^45^

In this study, preliminary phytochemical analysis showed that apart from cyclotides protein, the methylene chloride/methanol extract *O. affinis* (ODE) showed positive reactions for the presence of flavonoids, alkaloids and proteins, tannins, steroids and terpenoids. Flavonoids, alkaloids and proteins are most abundant constituents in ODE. Cyclotide-rich fraction (CRF) was found to contain flavonoids, alkaloids, saponins, proteins and steroids. Flavonoids and proteins were the most abundant constituents. In the acute toxicity studies in mice, the LD_50_ of both ODE and CRF was estimated to be greater than 5 g/kg. This signifies that at such high doses, these extract and fraction of *O. affinis* did not cause lethality nor produced signs of intoxication in the tests mice. A remote risk for possible acute intoxication is implied and thus they could be considered reasonably safe for all practical purposes.^24^

Malaria causes an acute systemic human disease that bears many similarities, both clinically and mechanistically, to those caused by other microbial (bacteria, rickettsia, and viruses) infections.^46^ Several researchers have shown or argued that most of the pathology seen in malaria infection, as well as other infectious diseases could be explained by activation of the inflammatory system, with the balance between the pro- and anti-inflammatory cytokines tipped in favour of the onset of systemic inflammation. 47–49

From the foregoing and on the strength of the traditional use of the herb in the treatment of malaria and malaria-related fever, it was necessary to investigate the extract and CRF for activities against acute and chronic inflammation in rats models as well as free radical scavenging potentials. In the study, group of rats administered both the extract and fraction of *O. affinis* showed remarkable anti-inflammatory potentials. Treatment with ODE and CRFsignificantly reduced carrageenan-induced paw oedema in rats compared to the untreated control. Carrageenan, a potent phlogistic agent, produces inflammation through the release of several inflammatory mediators. These chemical mediators produce increase in vascular permeability thus prompting fluid accumulation in tissues, which result in oedema.^50^,^51^ Hence, reduction in paw oedema may be due to inhibition of these inflammatory mediators by the treatment with ODE and CRF. The inhibition produced by these agents is slightly lower thanthe effect of the reference standard drug, piroxicam.

Similarly, the administration of ODE and CRF significantly attenuated progression of inflammation induced by formaldehyde in rat models compared to negative control. This effect was observed to be dose-dependent with the highest dose inhibition of chronic inflammation occurring at 400 mg ODE/kg. The cyclotide-rich fraction (CRF) also exhibited a similar effect as evidenced by its significant attenuation of the progression of oedema induced by formaldehyde in rat model of chronic inflammation at an extent slightly lower than the inhibition produced by the standard treatment of 1 mg dexamethasone/kg. Inhibition of formalin-induced pedal oedema in rats used as experimental chronic inflammation model is considered a suitable and reliable model to screen putative drug substances for chronic anti-inflammatory activity, as it closely resembles human arthritis.^52^

In a similar manner, in vitro anti-inflammatory assays showed that CRF inhibited lipopolysaccharide-induced released of pro-inflammatory mediators in cultures of RAW267.4 monocytic cells in a concentration-dependent manner. Inducible nitric oxide as well and TNF-α evoked by pretreatment of the macrophage cells were significantly (P<0.05) inhibited by treatment of 5–100 µg CRF/ml. These inhibitions are not likely due to cytotoxicity as the viability of the cells was observed to be greater than 96.87% at the concentrations of CRF used in the assay. There is now remarkably widespread acceptance that cytokines such as tumour necrosis factor (TNF) and interleukin-1 (IL-1) are the essential mechanism of systemic disease caused by infectious agents.^53^,^54^ High levels of these pro-inflammatory cytokines explain some of the notable symptoms of malaria disease such as anorexia, tiredness, aching joints and muscles, fever and sleepiness that patients experience in both *Plasmodium vivax* and *Plasmodium falciparum* malaria.^55^–^57^

The TNF-α and iNO oxide play a prominent role in host defence against variety of infections, including plasmodia infection. Excess production of iNO is associated with several diseases, including arthritis, autoimmune diseases, and septic shock and in several other chronic inflammatory diseases. The iNO is known to contribute to the inflammatory cascade by increasing vascular permeability and extravasations of fluids and proteins at inflammatory sites.^58^,^59^ Inhibition of high-output of NO production is thus a therapeutic strategy currently advocated for in the treatment of various inflammatory diseases. This study has demonstrated that the CRF is a potent inhibitor of TNF-a and iNO production in LPS-stimulated RAW264.7 macrophages.

In a recent contribution on the use of traditional herbal medicine in the treatment of malaria, it has been noted that inflammation is an important mechanism underlying the pathology of malaria and that most traditional therapy used by locals to treat malaria-related fever possess anti-inflammatory action.^60^

Treatment with CRF produced a significantly (P<0.05) lower level of reactive oxygen and reactive nitrogen species (ROS and RNS) in culture of RAW246.7 macrophages activated by PMA. Chemiluminescence of enhanced-luminol was used as an indicator for the production of ROS/RNS in macrophages activated by PMA as a stimulant of protein kinase C.^31^,^32^,^61^ The close associations between ROS and RNS and chronic inflammation are well documented. ROS induces chronic inflammation by the induction of COX-2, inflammatory cytokines (TNF-a, interleukin-1 (IL-1), IL-6), chemokines (IL-8, CXCR4) and pro-inflammatory transcription factors (NF-κB). [62–64] One of the many actions of the cytokines responsible for systemic inflammation is to disable oxidative phosphorylation within mitochondria. There is also a widely held consensus that infection with falciparum malaria is often fatal because mitochondria are unable to generate enough ATP to maintain normal cellular function.^65^,^66^ It is believed that apart from the sequestration of parasitized red cells that prevents sufficient oxygen getting to where it is needed, an equally important way ATP deficiency arises in malaria is an inability of mitochondria, through the effects of inflammatory cytokines on their function, to utilise available oxygen^67^,^68^ The reduced capacity of oxidative phosphorylation and concomitant ATP generation driven by pro-inflammatory cytokines released in malaria infection, as well as in other infectious diseases, explains the lethargy and tiredness associated with malaria infection.^69^

Apart from the beneficial anti-plasmodial activities recorded, the reactive radical(ROS/RNS)-scavenging activity and the anti-inflammatory activities of ODE and CRF are equally beneficial. These properties are relevant in the overall management and treatment of malaria and malaria fever. Complete isolation and characterization of the cyclotide was not achieved in the present study. A further study in which the cyclotides in *Odenlandia affinis* are isolated and completely characterized will be necessary in harnessing this unique and versatile cyclic protein as therapeutic option in a wide variety of ailments.

## Conclusion

This study showed that the dichloromethane/methanol extract and the cyclotide-rich fraction of the plant, Oldenlandia affinis showed anti-plasmodial activity against *Plasmodium berghei* using prophylactic and curative models in infected mice. Treatment with these extract and fraction also showed potent activities in acute and chronic rat models of inflammation which could be explained by the inhibition of expression and release of pro-inflammatory mediators (iNO and TNF-α) and reactive radical (ROS/RNS) scavenging activities witnessed in culture of macrophages in vitro. These findings could explain, at least in part, the successful use of the extract and decoctions from *Oldenlandia affinis* in the traditional treatment of malaria and malaria-associated fever.
